# The Possibility of Using Pine Bark Particles in the Chipboard Manufacturing Process

**DOI:** 10.3390/ma15165731

**Published:** 2022-08-19

**Authors:** Radosław Mirski, Adam Derkowski, Jakub Kawalerczyk, Dorota Dziurka, Joanna Walkiewicz

**Affiliations:** Department of Mechanical Wood Technology, Faculty of Forestry and Wood Technology, Poznan University of Life Sciences, 60-627 Poznan, Poland

**Keywords:** bark, chipboard, sawdust, wood-based material, wood chips

## Abstract

This research evaluated the possibility of using sawmill by-products from the roundwood-processing line in the production of wood-based panels. Due to its number of favorable properties, interesting chemical composition and large reserves resulting from the lack of industrial applications, the research focused particularly on the use of bark. Manufactured variants of boards differed in the proportions of wood chips to bark (70:30, 60:40, 50:50). Moreover, the boards containing only wood chips and a mixture of chips and sawdust were used as references. Urea-formaldehyde adhesive mixed with ammonium nitrate as a hardener was applied as a binding agent for the boards. Based on the mechanical properties (modulus of elasticity, modulus of rupture, internal bonding), physical properties (density, thickness swelling, water absorption) and content and emission of formaldehyde, it was found that it is possible to produce boards characterized by good properties from sawmill by-products without advanced processing. Moreover, the use of bark instead of sawdust in order to increase the homogeneity of the cross-section allows one to obtain panels with significantly lower formaldehyde emission and water uptake.

## 1. Introduction

In the production of sawn timber in the sawmill industry, up to 50% of the wood raw material initially intended for the production is so-called material loss [1]. In addition to losses resulting from the desorption changes, very large amounts of residues, such as wood chips, sawdust and bark, are generated in the production process [2,3]. Management of these by-products poses a great challenge [4]. Both the growing issue of shortages of available wood and the appearing trend of maximized use of renewable resources are leading to a constant increase in the application of wood processing residues, e.g., in the production of wood-based materials [5,6,7]. 

This paper is a continuation of research aimed at improvements in manufacturing boards characterized by high thickness and made of wood chips produced in the sawmill industry without processing them into particles. Mirski et al. [1] investigated the possibility of using wood chips and sawdust from primary wood processing lines as a raw material for the production of boards. Research has shown that the single-layer boards must have a density of 50–100 kg/m^3^ higher in order to reach appropriate mechanical properties required by the standards designed for particleboards as a result of using larger parties and obtaining an inhomogeneous cross-section. However, it was also found that the presence of outer layers (OL) made of microparticles resulted in a major improvement in the strength of the boards. The three-layer boards manufactured this way achieved properties roughly characteristic of P2-type boards. On the other hand, if the core layer (CL) was made of the mixture of chips and sawdust in a mass ratio of 70:30, the boards showed better properties compared to those produced only from the chips. According to the authors, sawdust filled the void spaces in the structure of the board and made it more homogeneous. Furthermore, it was also found that the chipboards in general are characterized by higher thickness swelling compared to both unfinished and laminated commercially available particleboards. The reason may be the incorporation of various additives, such as paraffin emulsion, in the industrial wood-based materials production process, which are not introduced to chipboard [8]. Moreover, it was also found that the properties of chip-sawdust boards depend also on the structure of the cross-section: the mass ratio of the core layer to outer layers, the dimensional fraction of the microparticles in the outer layers and the share of sawdust in the inner layer. The highest result of bending strength and the lowest value of thickness swelling was obtained for boards with the mass ratio of 60:40 (CL:OL), and the strongest internal bonding was noted for a variant characterized by the mass ratio of 70:30 [9]. In addition, research conducted by Mirski et al. [10] also showed that the application of pMDI (polymeric methylenediphenyl-4,4′-diisocyanate) as a binding agent allows for the use of sawmill by-products with high moisture content: 21% in the case of wood chips and 18% in the case of sawdust. 

The incorporation of bark particles in order to increase the homogeneity of the boards is an interesting concept. According to a paper published in 2017, the annual global amount of bark generated is estimated to be approximately 359,111,200 m^3^ [11], and a major industrial use has still not been found. It is mainly used as a source of energy in the combustion process and in horticulture (it maintains the moisture and lowers the pH of the soil) [12,13]. According to Sahin and Arslan [14], however, the continuing research on using bark in wood-based materials’ production can mitigate wood shortages. Bark particles were previously investigated for use as a filler for adhesives [15,16,17,18], as a substitute of wood particles in particleboard production [19,20,21,22], as a substitute for wood fibers [23] and as a basic material for decorative [24], sound-absorbing [25] and thermally insulating [11] panels. Research has shown that materials produced with the use of bark are characterized by good physical and mechanical properties. In this case, good properties mean the properties that they are comparable to those of panels made from raw materials usually intended for this purpose in industrial conditions (e.g., technical flour as a filler for adhesives or wood particles for the production of particleboards). Moreover, it was also found that they showed decreased formaldehyde emission. The reasons for the observed improvement include increased reactivity of adhesives due to the lowered pH, high content of phenolic substances that react with formaldehyde, etc. 

Therefore, the aim of the study was to investigate the possibility of incorporating ground bark particles characterized by an interesting chemical composition and great availability in the production of boards made of pine wood chips.

## 2. Materials and Methods

### 2.1. Materials 

**Figure 1:**
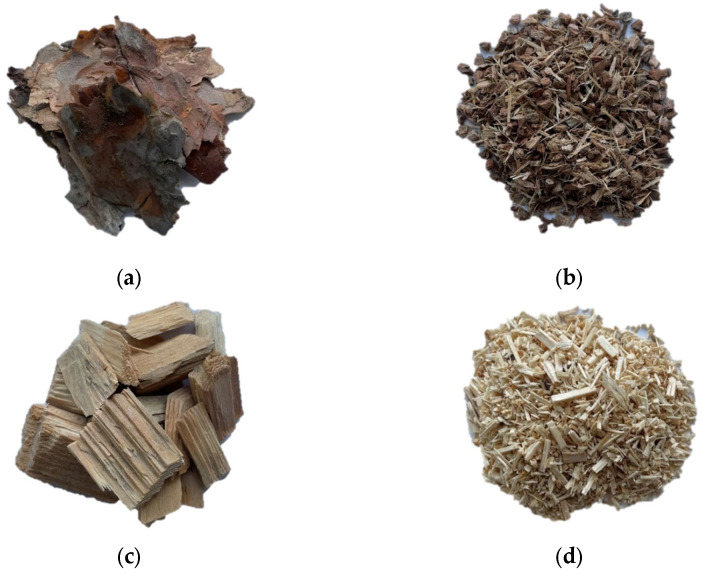
Materials used for the production of boards: (**a**) bark particles before grinding; (**b**) bark particles after grinding; (**c**) wood chips; (**d**) sawdust.

Raw materials such as chips, sawdust and bark (Figure 1) were provided from the pine (*Pinus sylvestris* L.) roundwood processing line in the sawmill Koszalińskie Przedsiębiorstwo Przemysłu Drzewnego (KPPD) Szczecinek S.A (Kalisz Pomorski, Poland), one of the largest producers of sawn wood in Poland. Chips were sieved through the sieve with the dimensions of 50 mm × 50 mm. Bark obtained during debarking of logs was ground three times in a disc chipper to obtain smaller particles. Urea formaldehyde (UF) adhesive was applied as a binding agent. It was provided by the industrial manufacturer of wood-based boards, and it was characterized by the following properties: viscosity of 470 mPa × s, gel time at 100 °C of 88 s, solid content of 58% and pH of 8.11. 

### 2.2. Characterization of the Materials 

**Figure 2:**
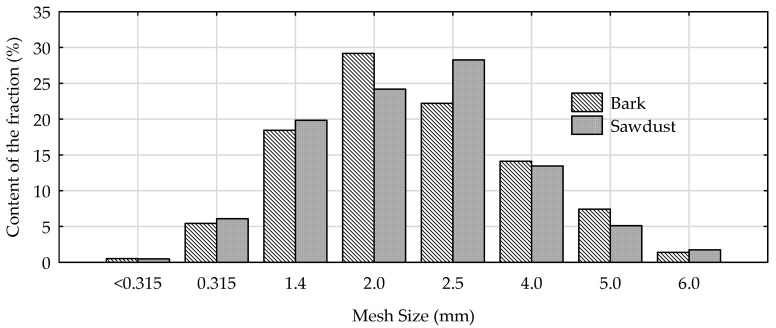
Fractional compositions of sawdust and bark.

In order to characterize the dimensions of the chips, 250 of them were measured using a caliper with an accuracy of 0.1 mm to determine length, width and thickness (Table 1). The fractional compositions of ground bark particles and sawdust were determined on the basis of sieve analysis with flat sieves made of mesh with the square perforations of: 6.3, 5.0, 4.0, 2.5, 2.0, 1.4, 0.315 mm. The results of conducted analysis are presented in Figure 2. 

Sawdust and bark (small particles) were characterized by very similar fractional compositions. The majority of the particles were in the range of 1.4 to 4.0 mm. 

### 2.3. Materials’ Preparation and Board Manufacturing 

**Figure 3:**
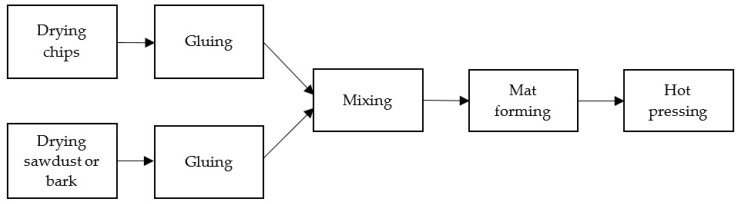
Schematic presentation of the board production process.

The steps of the board manufacturing process are presented in Figure 3. Materials were dried at 120 °C to reach a moisture content of 2 ± 2%. The gluing degree, which is a ratio of dry mass of the adhesive to the dry mass of lignocellulosic material, was 8% in the case of chips and 10% for sawdust and bark. An ammonium nitrate solution (20%) was introduced as a hardener to the gluing mixture to constitute 2% of the dry mass of the UF adhesive. The mat was formed manually. The hot pressing was conducted at 190 °C, with the unit pressure of 2.5 N/mm^2^ for 20 s/mm of the final board thickness. 

The compositions of variants of the manufactured boards with the assumed thickness of 20 mm and a density of 550 kg/m^3^ are shown in Table 2. 

### 2.4. Determination of Boards Properties 

Both the physical and mechanical properties of boards were tested in accordance with the relevant standards. Density was evaluated according to EN 323 [26]. The modulus of elasticity (MOE) and modulus of rupture (MOR) were investigated according to EN 310 [27]. Internal bonding (IB) was determined following the assumptions of EN 319 [28]. Moreover, thickness swelling (TS) in accordance with EN 317 [29] and water absorption (WA) were investigated after 2 or 24 h of soaking in water. WA was calculated based on Equation (1):

(1) $$\mathrm{WA}=\frac{m_{2}-m_{1}}{m_{1}}\times 100\%$$

where: m_1_ and m_2_ are the weights of sample before and after soaking, respectively. 

The investigations of physical and mechanical properties of the board were performed on 12 samples from each variant. The formaldehyde content (CF) was determined using the perforator method according to EN 120 [30]. Furthermore, the formaldehyde emission (EF) was investigated with the use of gas chamber analysis in accordance with EN ISO 12460-3 [31] using a GreCon GA 6000 analyzer (Fagus-GreCon Greten GmbH & Co. KG., Alfeld, Germany) content of formaldehyde in an aqueous solution was determined by spectrophotometry using the ammonium acetate and acetylacetone method. Absorbance of the samples was measured on a Biosens UV-5600 spectrophotometer (Biosens, Warsaw, Poland) at 412 nm. The results are expressed as the mean values of three replicates. 

### 2.5. Statistical Analysis 

The statistical analysis was performed with the use of STATISTICA 13.0 software. The differences between the variants were evaluated by one-way analysis of variance ANOVA followed by post hoc Tukey test with a significance level of α = 0.05. 

## 3. Results and Discussion 

The density of the board is one of the main factors determining its properties. It is well known that usually as the density increases, the mechanical properties also improve. The results of the investigations are shown in Table 3. 

**Figure 4:**
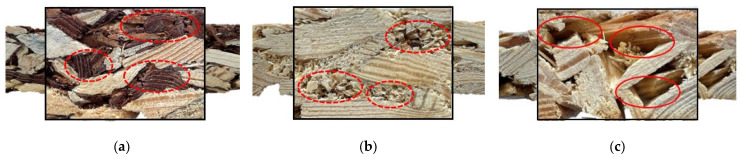
Cross-sections of the boards: (**a**) with bark particles (variant A); (**b**) with sawdust (variant D); (**c**) only with wood chips (variant E) (dashed circles indicate filled spaces; solid circles indicate void spaces).

The outcomes of statistical analysis show that there was a significant difference between the variants consisting of both wood chips and smaller particles and the variant made of only wood chips. Without the addition of bark or sawdust, boards were characterized by a density lower by approximately 20 kg/m^3^ (4%) than initially assumed. This was probably due to the void spaces in the board structure that were created between the chips. Their occurrence can be observed in Figure 4. However, both the amount of small particles and their type did not influence the results of density in a statistically significant way. The obtained values are very close to the assumed ones. In addition to affecting the density results, the presence of voids is disadvantageous because it can adversely affect the mechanical properties and water uptake of boards, and their degradation by microorganisms. 

**Figure 5:**
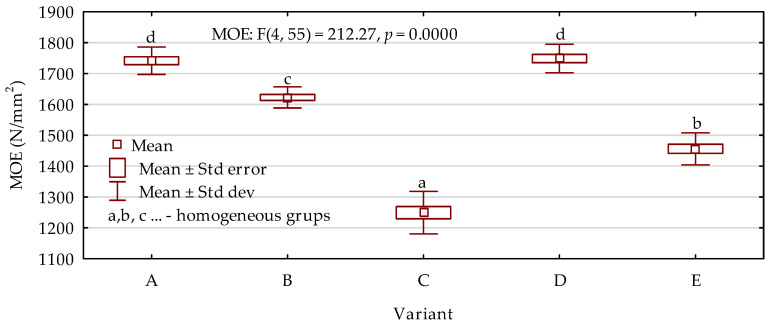
Modulus of elasticity results depending on the board variant (letters A–E mark variants of the boards).

**Figure 6:**
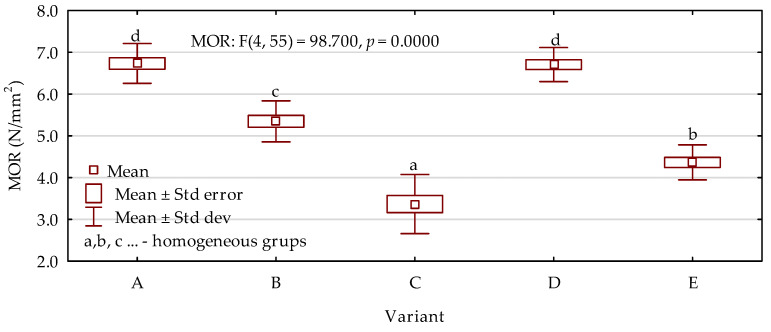
Modulus of rupture results depending on the board variant (letters A–E mark variants of the boards).

**Figure 7:**
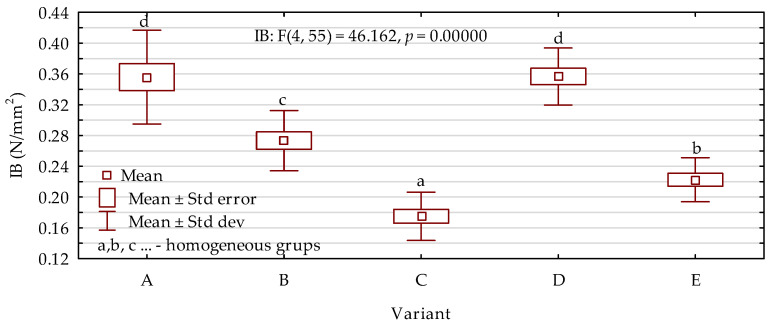
Internal bonding results depending on the board variant (letters A–E mark variants of the boards).

Results of the modulus of elasticity investigations are presented in Figure 5. Based on the outcomes, it was found that the highest MOE values were obtained for variants containing 30% small particles. In this case, the type of particles, whether they were bark or sawdust, did not have any statistically significant effect. The values were higher by approximately 17% in comparison with the boards consisting of only wood chips. A further increase in the amount of added small particles to 40% also resulted in the results of MOE being improved by 10% when compared with the chipboard. However, the poorest properties were observed when the share of bark increased to 50%. In this case, the MOE was 14% lower than in the case of the reference variant. Statistical analysis of the modulus of rupture results (Figure 6) showed exactly the same tendency as described for MOE. The best results were obtained for variants labeled as A and D with 30% filling particles incorporated to the manufacturing process. On the other hand, the lowest MOR was noted for boards containing a mixture of bark and wood chips at a weight ratio of 50:50. For the most advantageous variants, the results were higher by up to 35% than in the case of boards made of only wood chips. The worst variant, on the other hand, reached average values that were lower by 23%. The results of internal bonding were also in agreement with the previously described tendencies. The most favorable values noted were 38% higher than those of the reference boards with an inhomogeneous cross-section. When the share of bark was 50%, the results decreased significantly, by 18% (Figure 7).

Based on the outcomes of mechanical properties, it was found that mixing the wood chips with bark or sawdust in a weight ratio of 70:30 led to the production of boards with the best properties. This confirms the previous observations that the homogeneity of the cross-section considerably affects the strength parameters of boards [9]. The elimination of void spaces contributes to a more favorable distribution of stresses [32]. Moreover, the deterioration in mechanical properties of boards, which occurred in the variant assuming a 50% share of bark, could have result from its chemical composition. It is characterized by a significantly lower cellulose content than wood [33]. According to Baharoglu et al. [34], the use of lignocellulosic materials with lower cellulose contents can result in the manufacturing of boards characterized by lower strength parameters. Moreover, bark also contains a large amount of extractives. According to literature, it can also negatively affect the strength of glue bonds, which consequently could also influence the mechanical properties of the resultant boards [35,36]. 

Based on parameters such as thickness swelling and water absorption, the water resistance of boards was determined. The results are presented in Table 4.

The highest resistance was observed in the case of the chipboard manufactured without the addition of any smaller particles, and this was probably a result of lower density. However, the statistical analysis showed that the boards containing 40% and 50% bark were characterized by the same level of resistance. The reason for no statistically significant changes was probably the increase in the share of glue in the board (small particles had a higher gluing degree), which could have decreased the water uptake. The statistical analysis also showed that in the case of variants containing the same amounts of smaller particles (30%), boards produced with the use of bark were more resistant to water than sawdust-containing ones. This was probably the chemical composition of the material. Wood contains much a greater amount of holocellulose when compared to bark [33]. According to Baharoglu et al. [34], an increase in the share of hydrophilic components leads to increases in thickness swelling and water absorption of the boards. Moreover, the observed values were higher than the ones observed in previous studies regarding chipboard manufacturing [9]. Considering that the strength of the glue joints is one of the crucial factors affecting the water resistance of boards, the reason could be the binding agent used [37]. UF resin is characterized by a significantly lower resistance to water in comparison with the previously applied melamine-urea-formaldehyde (MUF) resin [38]. 

The emission of formaldehyde from UF resin-bonded wood-based materials has become a widely investigated problem. It is a highly reactive, colorless gas which can be responsible for serious human harm, especially in indoor environments. Formaldehyde has been classified by the International Agency for Research on Cancer as a “known human carcinogen,” and since then, the level of permissible emission has been gradually lowered [39,40]. Therefore, there are many ongoing studies focused on the reduction or even elimination of formaldehyde use in adhesives [41]. The results of investigations performed with the use of the perforator method and gas chamber analysis are presented in Table 5. 

Regardless of the method by which the analysis was carried out, the results showed the same trend. The lowest CF and EF were observed for variants made of only wood chips and chips mixed with bark in the ratio 70:30. Between them, no statistically significant difference was noted. However, it seems that the type of small particles affected the results. When comparing the boards containing the same amounts of wood (D) and bark (A), it was found that the use of bark reduced both the emission and content of formaldehyde. The reason was probably its chemical composition, especially the contents of tannins and phenolic compounds [20]. The majority of these substances are characterized by the ability to react with formaldehyde [42,43]. A similar effect was observed in the experiments regarding the use of bark as a filler for adhesives [15,17,18,33] and as a substitute for wood particles in boards [20,24]. Moreover, it was also found that bark has the ability to absorb formaldehyde from an aqueous solution [16] and from contaminated air [44]. However, a further increase in the share of bark particles to 40 or 50% resulted in a statistically significant increase in the formaldehyde emission. The reason was probably the higher gluing degree of smaller particles, and consequently, the higher amount of UF adhesive, which still remained the main source of formaldehyde in the boards. 

## 4. Conclusions

The wastes from the sawmill industry, such as chips, sawdust and bark, can be used as the materials for the production of boards characterized by good mechanical and physical properties. However, the application of UF resin instead of MUF resin for boards made of sawmill by-products results in significant increases in their thickness swelling and water absorption. Furthermore, their properties strongly depend on the proportions of individual components. The most advantageous properties were observed for variants consisting of a mixture of wood chips and smaller particles (sawdust or bark) in a weight ratio of 70:30 due to the homogeneous structure of boards. Moreover, the replacement of sawdust with bark allows one to produce materials with equally good mechanical properties, lower water uptake and decreased formaldehyde content and emission. A potential limitation that will be the subject of further research is the variability of the chemical composition of the bark. The variability of the results depending on the species, habitat, age, size and quality of the barked log will be examined.

## Figures and Tables

**Table 1:** Dimensions of wood chips.

Material	Length (mm)			Width (mm)			Thickness (mm)		
	Mean	Min.	Max.	Mean	Min.	Max.	Mean	Min.	Max.
Wood chips	33.9 (6.3)	18.4	54.7	14.5 (4.2)	7.3	28.7	5.1 (1.3)	1.9	9.9

Note: values in parentheses mean standard deviations; min. means minimum value; max. means maximum value.

**Table 2:** Variants of manufactured boards.

Variant Label	Share of Components in Boards (%)		
	Chips	Bark	Sawdust
A	70	30	0
B	60	40	0
C	50	50	0
D	70	0	30
E	100	0	0

**Table 3:** Density of manufactured boards.

Variant Label	Density of Boards (kg/m^3^)		
	Mean	Min.	Max.
A	551 (12.6) b	538	565
B	548 (11.4) b	537	561
C	549 (10.6) b	538	560
D	552 (11.1) b	541	563
E	531 (10.3) a	516	551

Note: Values in parentheses mean standard deviations; min. means minimum value; max. means maximum value; letters a,b indicate the homogeneous groups.

**Table 4:** Thickness swelling and water absorption of boards depending on the variant.

Variant Label	Thickness Swelling (%)		Water Absorption (%)	
	2 h	24 h	2 h	24 h
A	16.3 (0.6) b	18.9 (0.4) b	92.9 (0.8) b	93.7 (0.6) b
B	15.7 (0.9) ab	18.5 (0.7) ab	91.7 (0.9) ab	92.9 (0.7) ab
C	15.9 (1.1) ab	18.4 (0.9) ab	91.3 (1.3) ab	93.2 (1.1) ab
D	17.2 (0.4) c	19.8 (0.6) c	94.6 (0.6) c	95.9 (1.0) c
E	15.2 (0.7) a	17.6 (0.6) a	87.5 (2.4) a	91.2 (1.3) a

Note: Values in parentheses mean standard deviations; letters a,b,c indicate the homogeneous groups.

**Table 5:** Formaldehyde emission and content depending on the variant.

Variant Label	Formaldehyde Content (mg/100 g)	Formaldehyde Emission (mg/m^2^ h)
A	3.3 (0.3) a	2.1 (0.3) a
B	5.1 (0.2) c	4.6 (0.3) c
C	5.4 (0.3) c	4.4 (0.2) c
D	4.3 (0.2) b	3.1 (0.3) b
E	3.1 (0.4) a	2.3 (0.2) a

Note: Values in parentheses mean standard deviations; letters a,b,c indicate the homogeneous groups.

## Data Availability

The data presented in this study are available on request from the corresponding author.
